# ERRATUM: Organizational culture and climate for patient safety in Intensive Care Units

**DOI:** 10.1590/1980-220X-REEUSP-2024-ER17en

**Published:** 2024-10-04

In the article “Organizational culture and climate for patient safety in Intensive Care Units”, with the DOI: https://doi.org/10.1590/S0080-623420150000700018, published by the journal: “Revista da Escola de Enfermagem da USP, volume 49 (spe) of 2015, p. 121–127, on page 127:

**Now read:**

## Financial support

This study was financed in part by the Coordenação de Aperfeiçoamento de Pessoal de Nível Superior – Brasil (CAPES) – Finance Code 001.

