# Supplementary material for: ERRATUM: Organizational culture and climate for patient safety in Intensive Care Units
Source: Rev Esc Enferm USP. 2024 Oct 4;58:e2024ER17. doi: 10.1590/1980-220X-REEUSP-2024-ER17en (PMC11460652; doi:10.1590/1980-220X-REEUSP-2024-ER17en)
Supplement: Translation

# Translation: ERRATA: Cultura e clima organizacional para segurança do paciente em Unidades de Terapia Intensiva

**DOI:** 10.1590/1980-220X-REEUSP-2024-ER17pt

No artigo “Cultura e clima organizacional para segurança do paciente em Unidades de Terapia Intensiva”, com número de: DOI: https://doi.org/10.1590/S0080-623420150000700018, publicado na Revista da Escola de Enfermagem da USP, volume 49 (spe) de 2015, p. 123–130, na página 130:

**Leia-se:**

## Apoio financeiro

O presente trabalho foi realizado com apoio da Coordenação e Aperfeiçoamento de Pessoal de Nível Superior – Brasil (CAPES) – Código de Financiamento 001.
